# Simulation of Carbon Dioxide Absorption in a Hollow Fiber Membrane Contactor Under Non-Isothermal Conditions

**DOI:** 10.3390/membranes15030093

**Published:** 2025-03-14

**Authors:** Youkang Jin, Lei Wang, Jinpeng Bi, Wei Zhao, Hui Zhang, Yuexia Lv, Xi Chen

**Affiliations:** 1 School of Mechanical Engineering, Qilu University of Technology (Shandong Academy of Sciences), Jinan 250353, China202101010060@stu.qlu.edu.cn (X.C.); 2 Shandong Institute of Mechanical Design and Research, Jinan 250031, China; 3 China Coal Society, Beijing 100013, China

**Keywords:** membrane gas absorption, CO_2_ separation, computational simulation, non-isothermal

## Abstract

CO_2_ capture by membrane gas absorption technology has been considered a promising alternative to mitigate or stabilize atmospheric CO_2_ concentrations. The non-isothermal nature of the CO_2_ absorption process in hollow fiber membrane contactors is a critical factor that significantly influences CO_2_ removal performance. In the present study, a non-isothermal mathematical model and a two-dimensional computational simulation were carried out to evaluate the CO_2_ separation by three typical absorbents in a polyvinylidene fluoride hollow fiber membrane contactor under non-wetting operation mode. The simulation results exhibited good matching with the published experimental data with the deviations in the range of lower than 5%, which validated the reliability of the developed numerical model. A significant temperature increase ranging from 2 to 15 K was observed along the length of the hollow fiber membrane contactor, which further facilitated the absorption and reaction process in this study. The results showed that potassium glycinate exhibited the highest absorption capacity, followed by monoethanolamine and 1-ethyl-3-methylimidazolium. In addition, the mass transfer could be enhanced by increasing the liquid flow rate, absorbent concentration, module length, and membrane porosity, while increasing the gas velocity and CO_2_ inlet concentration were unfavorable for the CO_2_ removal process.

## 1. Introduction

The carbon dioxide generated during the combustion process of fossil fuels is a major contributor to greenhouse gas emissions. The resulting global warming has brought severe challenges to global food security, water resources, ecological systems, energy supplies, human health, and large-scale infrastructure. To address this issue, it is particularly imperative to implement effective measures to limit CO_2_ emission from large-scale industrial sources [1]. According to the Energy Technology Perspectives 2020 report issued by the International Energy Agency [2], renewable energy generation, bioenergy, hydrogen energy, and carbon dioxide capture and storage have been identified as key technologies to achieve global net zero emissions. Among them, carbon dioxide capture and storage is the only technology which can directly stabilize or even reduce the atmospheric carbon dioxide concentrations in the short term.

The CO_2_ post-combustion capture system is currently the most advanced technology to capture CO_2_ from the flue gas of coal-fired power plants or other large CO_2_ emission sources without the significant retrofitting of existing infrastructure. Chemical absorption is the most well-established technology and has been extensively utilized in the gas separation industry for decades. However, conventional gas absorption towers and scrubbers are generally encountered with challenges of flooding, absorbent losses, entrainment, liquid channeling, foaming, and other operation problems [3]. The applications of another promising membrane gas separation technology are generally limited by the tradeoff between permeability and selectivity, even though it has the advantages of a high specific surface area, flexible design, and compact and simple structure [4]. Consequently, researchers have extensively explored the integration of two or more CO_2_ separation technologies, with the aim of enhancing the CO_2_ removal efficiency and eliminating the operational challenges.

Membrane gas absorption technology is a hybrid process that combines the advantages of gas absorption technology with membrane separation technology, which has been considered a promising approach in the field of CO_2_ separation [5,6]. Compared with conventional absorption towers or columns, the absorbent and flue gas in a hollow fiber membrane contactor flow in the tube side and shell side, respectively, thus avoiding the operational problems such as flooding, channeling, entrainment, and foaming. Furthermore, membrane gas absorption technology has the advantages of a compact structure, large specific surface area, good operational flexibility, modular design, low energy consumption, and easy linear scaling [7]. The main drawback of membrane gas absorption technology is the membrane wetting over long-time operation, which can be prohibited by the superhydrophobic surface modification of porous polymer membranes [8]. Unlike traditional membrane gas separation, in this process, the hollow fiber membrane does not provide CO_2_ selectivity but rather serves as the contact interface between the gas and liquid phases. The selectivity is provided by the absorbent flowing on the tube side of the membrane contactor. Therefore, the selection of an absorbent is a critical factor in improving CO_2_ removal efficiency and reducing the energy consumption. Alkanolamines, pure alkaline solutions, amino acid salts, and ionic liquids have been extensively used as the absorbents for CO_2_ removal by the hollow fiber membrane contactors [9,10,11].

Mathematical modeling and simulation are crucial methods to predict the mass transfer performance and CO_2_ removal efficiency, which plays an important role in the design and scale-up of the CO_2_ separation process by the membrane contactor. A variety of research projects have been carried out to investigate the influences of different absorbents and operating parameters on CO_2_ removal efficiency based on isothermal mathematical models under the non-wetting mode. Nakhjiri et al. [12,13,14,15] compared the effects of different liquid absorbents on CO_2_ separation in polyvinylidene fluoride (PVDF) and polypropylene (PP) hollow fiber membrane contactors. The numerical results showed that CO_2_ concentration at the outlet was reduced from 4 mol/m^3^ to 0.76, 0.45 and 0.71 mol/m^3^ using ethylenediamine, (piperazinyl-1)-2-ethylamine, and polystyrene as absorbents, respectively. Compared with NaOH and triethylamine, monoethanolamine (MEA) showed better efficiency in removing CO_2_, and 2-tert-butylaminoethanol can achieve a decarbonization effect similar to that of MEA [16]. The addition of nanoparticles can enhance Brownian motion and grazing effects. Ghasem et al. [17] developed a two-dimensional mathematical model to simulate the CO_2_ absorption process of water-based nanofluids enhanced by carbon nanotubes in hollow fiber membrane contactors. The simulation results showed that the CO_2_ removal rate increased by about 20% after adding 0.5 wt. % carbon nanotubes. Furthermore, the addition of montmorillonite nanoparticles and SiO_2_ nanoparticles can also improve the CO_2_ removal rate [18,19]. Vaezi et al. [20] and Bozonc et al. [21] proposed a two-dimensional mathematical model to evaluate the CO_2_ removal efficiency in a porous hydrophobic polytetrafluoroethylene and PP hollow fiber membrane using 3-diethylaminopropylamine and MEA as the absorbent. The influences of amine concentration, liquid and gas flow rate, liquid temperature, CO_2_ partial pressure, membrane tortuosity, packing density, and hollow fiber numbers on CO_2_ absorption performance were studied. Xia et al. [22] developed a two-dimensional mass transfer model to compare the CO_2_ absorption by ammonia, MEA, and diethanolamine under different operating conditions and membrane geometric properties. Bozonc et al. [23] simulated the CO_2_ absorption process using MEA in a hollow fiber membrane contactor and studied the effects of operating parameters on the removal process. All above simulation models have been developed under isothermal conditions for simplification. However, the absorption process of CO_2_ by absorbents is non-isothermal and exothermic, during which the reaction heat is accumulated as the absorbent moves toward the outlet. The non-isothermal process definitely influences the subsequent CO_2_ absorption process, and a non-isothermal model shall be developed to present a more realistic approach that is relevant to the CO_2_ absorption by reactive absorbents in hollow fiber membrane contactors.

There have been very limited non-isothermal models to estimate the influences of temperature variations on reaction kinetics. Sohaib et al. [24] developed a two-dimensional non-isothermal model to investigate the non-isothermal behaviors of the CO_2_ absorption process and its influences on the reaction kinetics, by using four amino acid-based ionic liquids in a hydrophobic polypropylene membrane contactor. A 10–25 K temperature increase was observed along the membrane contactor length, which was attributed to the release of dissolution reaction energy, which further affected relevant parameters including diffusive solubility, rate constants, and reaction rates. The simulation results of Zaidiza et al. [25] also found that, the temperature profiles along the membrane contactor were increased by nearly 30 K, which further enhanced the reaction rate of MEA with CO_2_ by 5%. Evidently, the non-isothermal nature of the CO_2_ absorption process should be taken into account to evaluate the CO_2_ removal performance using absorbents in hollow fiber membrane contactors. Therefore, it is correspondingly the interest of the present study to develop a two-dimensional non-isothermal model, with the aim of providing a more realistic approach towards the CO_2_ absorption process using three typical absorbents in a PVDF hollow fiber membrane contactor. Potassium glycinate (PG), MEA, and 1-ethyl-3-methylimidazolium acetate ([EMIM][Ac]) were selected as the absorbents to represent the amino acid salt, amine solution, and ionic liquid, respectively. Furthermore, the influences of various operational parameters and hollow fiber module specifications on CO_2_ removal performance were studied using the proposed model.

## 2. Methodology

### 2.1. Model Development

In this study, a two-dimensional finite element mathematical model was developed to simulate the non-isothermal CO_2_ separation process from flue gas using three typical absorbents in a hollow fiber membrane contactor operating under a non-wetted mode. COMSOL software version 6.1 was employed to evaluate the CO_2_ removal efficiency and concentration distribution within the gas–liquid membrane contactor. PVDF was selected as the hydrophobic porous membrane, mainly due to its intrinsic hydrophobicity, affordability, and exceptional thermal and chemical stability. Three representative aqueous solutions representing alkanolamines, amino acid salts, and ionic liquids were selected as the chemical absorbents, named MEA, PG, and [EMIM][Ac], respectively.

**Figure 1:**
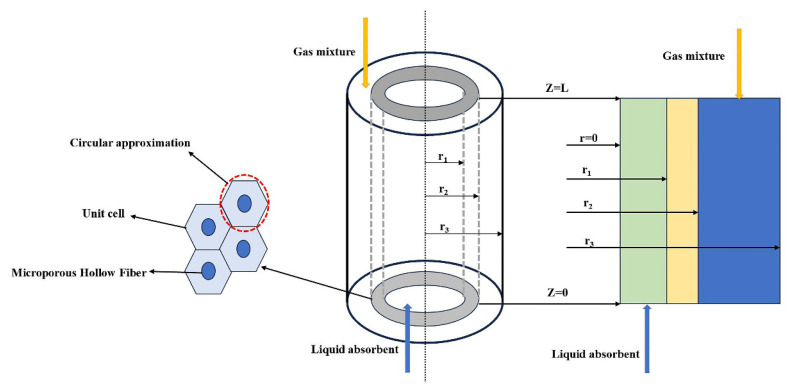
Schematic diagram of CO_2_ absorption in a hollow fiber membrane contactor.

The schematic diagram of the hollow fiber membrane contactor used for model development is presented in Figure 1. The flue gas composed of CO_2_ and N_2_ enters the shell side of the hollow fiber membrane contactor from the top (Z = L) and flows downward, while the absorbent enters the tube side from the bottom (Z = 0), flowing in the opposite direction. Driven by the concentration gradient, CO_2_ in the flue gas mixture diffuses through the membrane pores to the gas–liquid contact interface, and it is further absorbed into the liquid phase by the absorbent. The model in this study is established based on the Happel free surface model, which had been extensively used for hollow fiber membrane contactors [26]. This model considers only a portion of the fluid surrounding the hollow fibers, approximating it as an annular cross section.

The model is developed based on the following assumptions: the CO_2_ removal process occurs under steady-state conditions, with both the gas flowing in the shell side and liquid flowing in the tube side being laminar; Henry’s Law is applied to determine the gas–liquid equilibrium at the interfacial boundary. The gas phase on the shell side is treated as an ideal gas.

The specifications for the PVDF hollow fiber membrane contactor used in this study are detailed in Table 1. The simulations were conducted at a temperature of 303.15 K and a pressure of 0.1 MPa.

### 2.2. Governing Equations and Boundary Conditions

The continuity equation for the component *i* can be expressed as follows [27]:

(1) $$\frac{\partial C_{i}}{\partial t}=-D_{i}\nabla^{2}C_{i}-{\nabla C}_{i}V_{Z}+R_{i}$$

where *C_i_* is the concentration, mol/m^3^; *D_i_* is the diffusion coefficient, m^2^/s; *R_i_* is the reaction rate of component *i* along the axial direction, and the reaction only occurs in the liquid phase, mol/m^3^·s; *V_Z_* is the axial velocity, m/s; and *t* is time, s.

#### 2.2.1. Shell Side Equations

The continuity equation for the gas side in the shell compartment under a steady state can be expressed as follows [28]:

(2) $$D_{{CO}_{2},shell}\left[\frac{\partial^{2}C_{{CO}_{2},shell}}{\partial r^{2}}+\frac{1}{r}\frac{\partial C_{{CO}_{2},shell}}{\partial r}+\frac{\partial^{2}C_{{CO}_{2},shell}}{\partial Z^{2}}\right]=V_{Z,shell}\frac{\partial C_{{CO}_{2},shell}}{\partial Z}$$

where *D_CO2,shell_* is the diffusion coefficient of component CO_2_, m^2^/s; *C_CO2,__shell_* is the concentration of component CO_2_ in the shell compartment, mol/m^3^; and *V_Z,shell_* is the velocity in the shell compartment, m/s.

When the gas phase is assumed under a laminar state, the velocity distribution of the gas phase in the shell side can be calculated based on the Happel model, which can be expressed by the following [29]:

(3) $$2\bar{V}\left[1-{\left(\frac{r_{2}}{r_{3}}\right)}^{2}\right]\times \left[\frac{{\left(r/r_{3}\right)}^{2}-{\left(r_{2}/r_{3}\right)}^{2}+2\ln\left(r_{2}/r\right)}{3+{\left(r_{2}/r_{3}\right)}^{4}-4{\left(r_{2}/r_{3}\right)}^{2}+4\ln\left(r_{2}/r_{3}\right)}\right]=V_{Z-shell}$$

where $\bar{V}$ is the shell-side average velocity, m/s; *r*_2_ is the hollow fiber outer diameter, mm; *r*_3_ is the effective radius of the shell side, which can be expressed by the following [30]:

(4) $$r_{3}=r_{2}{\left(\frac{1}{1-\varphi }\right)}^{\frac{1}{2}}$$

where *φ* is the volume fraction of the void, expressed by the following Equation [12]:

(5) $$1-\varphi =n{\left(\frac{r_{2}}{R}\right)}^{2}$$

where *n* is the number of hollow fibers and *R* is the inner diameter of the module, mm.

The thermal balance equation in the shell compartment is presented as follows:

(6) $$\lambda_{g,shell}\left[\frac{\partial^{2}T_{g,shell}}{\partial r^{2}}+\frac{1}{r}\frac{\partial T_{g,shell}}{\partial r}+\frac{\partial^{2}T_{g,shell}}{\partial Z^{2}}\right]=V_{Z-shell}C_{p,g}\rho_{g}\frac{\partial T_{g,shell}}{\partial Z}$$

where $\lambda_{g,shell}$ is the thermal conductivity of the gas phase in the shell compartment, W/m·K; *T_g,shell_* is the temperature of the gas phase in the shell compartment, K; *C_p,g_* is the specific heat of gas phase in the shell compartment, J/mol·K.

#### 2.2.2. Membrane Side Equations

When the membrane pores are filled with gas, the mass transfer resistance of the membrane is typically neglected, which corresponds to the non-wetting operation mode considered in this study. Under these conditions, CO_2_ diffuses from the outer surface of the hollow fiber to the gas–liquid contact interface through the microporous pores. Thus, the steady-state continuity equation within the hollow fiber membrane can be expressed as follows [18]:

(7) $$D_{{CO}_{2},mem}\left[\frac{\partial^{2}C_{{CO}_{2},mem}}{\partial r^{2}}+\frac{1}{r}\frac{\partial C_{{CO}_{2},mem}}{\partial r}+\frac{\partial^{2}C_{{CO}_{2},mem}}{\partial Z^{2}}\right]=0$$

where *D_CO2,mem_* is the diffusion coefficient of CO_2_ within the membrane pores, m^2^/s; *C_CO2,mem_* is the concentration of CO_2_ inside the membrane pores, mol/m^3^. The diffusion coefficient of the CO_2_ inside the membrane pores is influenced by the membrane tortuosity and porosity. The diffusion coefficient of CO_2_ inside the membrane pores can be expressed as follows [31,32]:

(8) $$D_{{CO}_{2},mem}=\frac{D_{{CO}_{2},shell}\times \varepsilon }{\tau }$$

The thermal balance equation of CO_2_ at the membrane side can be presented by the following:

(9) $$\lambda_{{CO}_{2},mem}\left[\frac{\partial^{2}T_{{CO}_{2},mem}}{\partial r^{2}}+\frac{1}{r}\frac{\partial T_{{CO}_{2},mem}}{\partial r}+\frac{\partial^{2}T_{{CO}_{2},mem}}{\partial Z^{2}}\right]=0$$

(10) $$\lambda_{{CO}_{2},mem}=\varepsilon \lambda_{{CO}_{2},shell}+\left(1-\varepsilon \right)\lambda_{mem}$$

where $\lambda_{{CO}_{2},mem}$ is the thermal conductivity of CO_2_ within the membrane pores, W/m·K; $\lambda_{mem}$ is the thermal conductivity of membrane material, W/m·K; *T_CO2,mem_* is the temperature of CO_2_ within the membrane pores, K; and *ε* is the membrane porosity.

#### 2.2.3. Tube Side Equations

The steady-state continuity equation for CO_2_ in the tube compartment consists of convection, diffusion, and chemical reaction, which can be expressed by the following [33]:

(11) $$D_{{CO}_{2},tube}\left[\frac{\partial^{2}C_{{CO}_{2},tube}}{\partial r^{2}}+\frac{1}{r}\frac{\partial C_{{CO}_{2},tube}}{\partial r}+\frac{\partial^{2}C_{{CO}_{2},tube}}{\partial Z^{2}}\right]\;\;\;\;\;\;=V_{Z,tube}\frac{\partial C_{{CO}_{2},tube}}{\partial Z}-R_{{CO}_{2},tube}$$

where *D_CO2,tube_* is the CO_2_ diffusion coefficient in the tube compartment, m^2^/s; *C_CO2,tube_* is the CO_2_ axial concentration in the tube compartment, mol/m^3^; *V_Z,tube_* is the CO_2_ axial velocity in the tube compartment, m/s; and $R_{{CO}_{2},tube}$ is the reaction rate of CO_2_ in the liquid phase in the tube side, mol/m^3^·s.

Since radial convection is significantly lower than axial convection, only axial convection is considered in the model. The velocity distribution of the laminar flow in the tube is denoted by the following [30]:

(12) $$V_{Z,tube}=2v\left[1-{\left(\frac{r}{r_{1}}\right)}^{2}\right]$$

where *v* is the liquid velocity at the inlet, m/s.

The thermal balance equation of CO_2_ in the tube compartment is presented as follows:

(13) $$\lambda_{L,tube}\left[\frac{\partial^{2}T_{L,tube}}{\partial r^{2}}+\frac{1}{r}\frac{\partial T_{L,tube}}{\partial r}+\frac{\partial^{2}T_{L,tube}}{\partial Z^{2}}\right]+R_{L,tube}∆H_{abs}\;\;\;=V_{Z,tube}C_{p,L}\rho_{L}\frac{\partial T_{L,tube}}{\partial Z}$$

where $\lambda_{L,tube}$ is the thermal conductivity of the liquid phase in the tube side, W/m·K; *T_L,tube_* is the temperature of the liquid phase in the tube side, K; $∆H_{abs}$ is the enthalpy change of the absorption process, J/mol; *C_p,__L_* is the liquid phase specific heat in the tube compartment, J/mol·K; and $R_{L,tube}$ is the reaction rate of absorbent in the tube side, mol/m^3^·s.

#### 2.2.4. Boundary Conditions

According to the research results obtained by Lee et al. [34], the effect of temperature on the absorbent performance is negligible in the temperature range of 293.15 K to 323.15 K. In this study, parameter values at 303.15 K are used in the simulation. Table 2, Table 3 and Table 4 provide the corresponding boundary conditions applied in the simulation for the shell side, membrane side, and tube side, respectively. These boundary conditions are crucial for accurately modeling the CO_2_ separation process within the hollow fiber membrane contactor. The partition coefficient *m* is defined as the ratio of the CO_2_ concentration in the liquid phase to that in the gas phase at the interface. In the simulation, the value of *m* is usually considered as a constant value.

### 2.3. Reaction Mechanism

In the MEA-CO_2_-H_2_O system, the reaction between MEA and CO_2_ is described by a zwitterionic mechanism, which proceeds by forming zwitterions as intermediates.

(14) $${CO}_{2}+RNH_{2}\to {RNH}_{2}^{+}{COO}^{-}$$

The intermediate zwitterion loses a proton by reacting with the MEA molecule.

(15) $${RNH}_{2}^{+}{COO}^{-}+RNH_{2}\to {RNHCOO}^{-}+{RNH}_{3}^{+}$$

Therefore, the total reaction between MEA and CO_2_ is as follows:

(16) $${CO}_{2}+2RNH_{2}\to {RNHCOO}^{-}+{RNH}_{3}^{+}$$

The reaction between the amino acid salt PG solution and CO_2_ is also described by the zwitterion mechanism. The formation of zwitterions follows the following reaction:

(17) $$R_{2}NH+{CO}_{2}↔+R_{2}N^{+}HCO_{2}^{-}$$

The deprotonation of zwitterions by bases also follows the following reaction:

(18) $$R_{2}N^{+}HCO_{2}^{-}+B_{i}\to R_{2}NCO_{2}^{-}+B_{i}H^{+}$$

The chemisorption mechanism is proposed by Gurau et al. The basic chemistry of [Emim][Ac] IL and CO_2_ is described by the following reversible reaction:

(19) $$2\left[C_{2}min\right]{\left[CH_{3}^{+}COO\right]}^{-}↔\left[C_{2}min\right]+\left[C_{2}minH{\left(CH_{3}^{+}COO\right)}_{2}^{-}\right]$$

(20) $$\left[C_{2}min\right]+{CO}_{2}\to \left[{C_{2}min}^{+}-{COO}^{-}\right]$$

The CO_2_ removal efficiency is a critical indicator for evaluating the separation performance of the membrane contactor. It can be expressed by the following equation:

(21) $$\eta =100\times \left(1-\frac{C_{out}}{C_{in}}\right)$$

where *C_in_* and *C_out_* are the concentration of CO_2_ at the inlet and outlet of the membrane contactor, respectively, mol/m^3^.

### 2.4. Verification of Grid Independence

**Figure 2:**
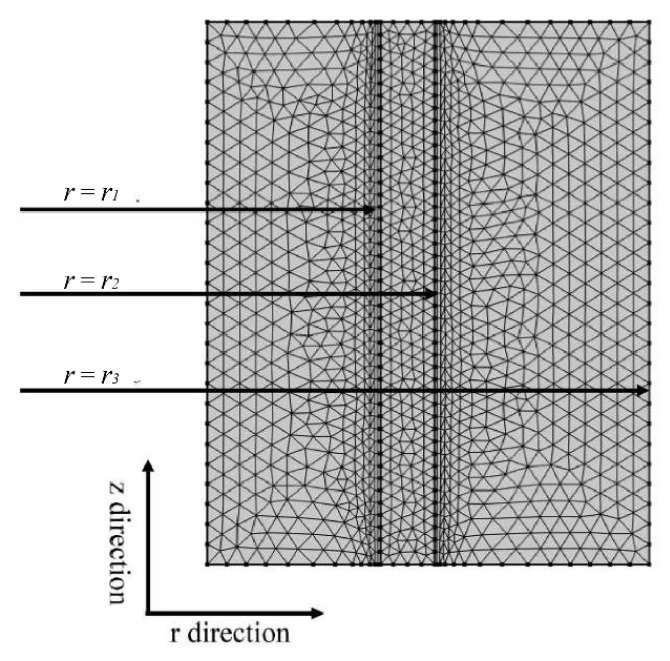
Triangular mesh division of shell, membrane, and tube sides in HFMC.

Figure 2 illustrates the triangular meshing technique used to analyze the behavior of gas and liquid solutions within a microporous hollow fiber membrane contactor. The PARDISO solver, known for its memory efficiency, was employed to enhance the computational efficiency and accuracy. A finer mesh size and higher degree of aggregation near the membrane wall were utilized to capture the reactions and gas–liquid interactions occurring within the membrane pores, thereby improving the fidelity of the mathematical model and reducing computational discrepancies. It is important to note that increasing the number of grids can reduce computational errors and improve accuracy. However, this also results in a longer computational time. Therefore, an optimum mesh grid number must be determined.

**Figure 3:**
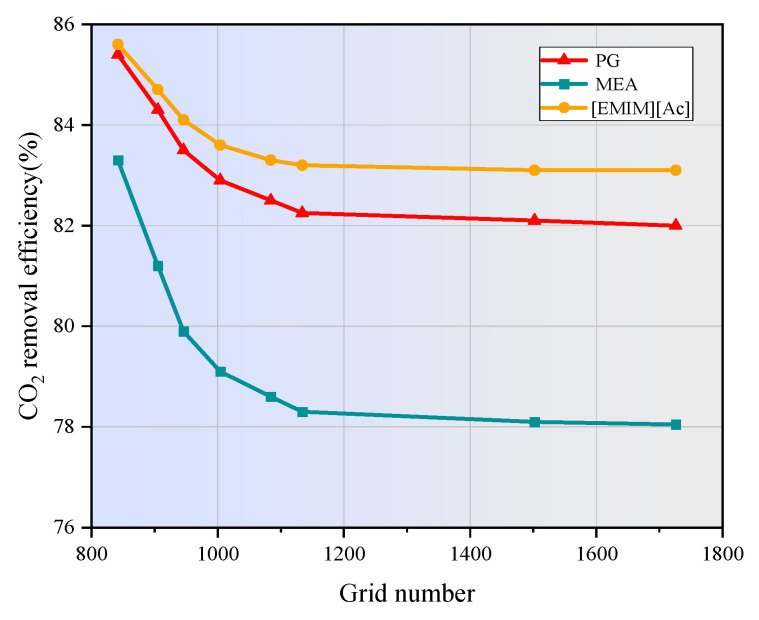
Effect of grid number on CO_2_ removal efficiency.

Figure 3 shows the effect of the number of grids on the CO_2_ concentration at the shell side outlet. As depicted, the number of grids has a significant impact on simulation results when increasing from 800 to 1134. However, beyond 1134 grids, further increases have no noticeable effect on the results, indicating that the calculation accuracy becomes independent of the number of grids at this point. Therefore, to balance accuracy and computational cost, the number of grids used in this study were determined to be 1134.

### 2.5. Numerical Model Validation

**Figure 4:**
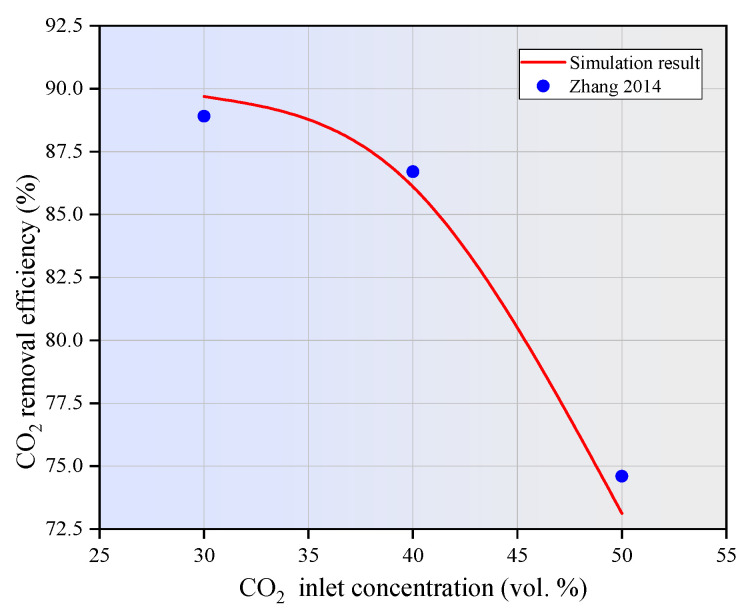
Comparison between simulation results and experimental data [40]. *C_L_ =* 10 wt.%, *V_g_* = 0.32 m/s, *V_L_* = 0.06 m/s, *T* = 298.15 K, *p* = 0.1 MPa.

To validate the reliability of the developed numerical model, the numerical results of CO_2_ removal efficiency using MEA in a PVDF hollow fiber membrane contactor under non-wetting mode were compared with the experimental results published in the literature [40]. As shown in Figure 4, the numerical simulation results exhibit a strong correlation with the experimental data, with an average error margin of less than 5%. The observed deviations between the experimental and simulation results are likely attributable to the estimation of certain constants and reaction kinetics during the development of the mathematical model. These findings suggest that the numerical method developed in this study is both reliable and accurate to predict the performance of the hollow fiber membrane contactor.

## 3. Results and Discussion

### 3.1. CO_2_ Concentration Distribution in the Shell Side

**Figure 5:**
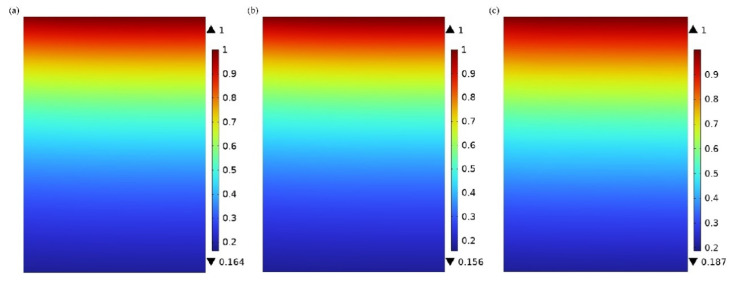
Concentration distribution of CO_2_ in the shell side: (**a**) MEA; (**b**) PG; and (**c**) [EMIM][Ac]. *C_CO2_* = 1 mol/m^3^, *C_L_* = 1600 mol/m^3^, *V_g_* = 0.4 m/s, *V_L_* = 1 m/s, *T* = 303.15 K, *p* = 0.1 MPa.

The CO_2_ concentration distribution in the shell side of the hollow fiber membrane contactor is presented in Figure 5. When the mixture gas of CO_2_ and N_2_ is introduced into the shell side from the top the hollow fiber membrane contactor at Z = L, the CO_2_ concentration in the shell side reaches its maximum value. It is assumed that the CO_2_ concentration in the tube side is zero at Z = 0. As the flue gas flows through the shell side, the mass transfer of CO_2_ is initially influenced by axial convection, driven by the laminar gas flow that carries CO_2_ downward at a certain velocity. Concurrently, CO_2_ molecules diffuse towards the hollow fiber membrane surface through molecular diffusion driven by the concentration gradient, as described by Fick’s Law. As depicted in Figure 5, the CO_2_ concentration gradually decreases in both the axial and radial directions as the gas moves toward the outlet. This decrease is attributed to the comprehensive effects of convective transport, molecular diffusion towards the membrane pores, permeation through the membrane pores, and subsequent reaction with the absorbent flowing countercurrent in the tube side. Under identical operating conditions, the highest axial variation in CO_2_ concentration is observed when PG is used as the absorbent, followed by MEA, with [EMIM][Ac] exhibiting the least variation. This trend corresponds to the relative CO_2_ absorption capacities of the absorbents, with PG having the highest absorption capacity, followed by MEA, and [EMIM][Ac] showing the lowest capacity.

### 3.2. Absorbent Concentration Distribution in the Tube Side

**Figure 6:**
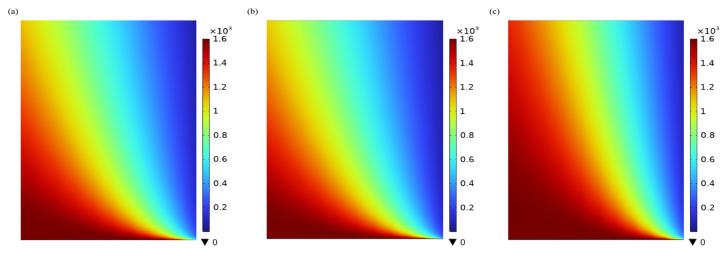
Concentration distribution of (**a**) MEA; (**b**) PG; and (**c**) [EMIM][Ac]. *C_CO2_* = 1 mol/m^3^, *C_L_* = 1600 mol/m^3^, *V_g_* = 0.4 m/s, *V_L_* = 1 m/s, *T* = 303.15 K, *p* = 0.1 MPa.

Figure 6 shows the axial concentration distributions of MEA, PG, and [EMIM][Ac] in the tube side of the membrane contactor operating under the non-wetting mode. As CO_2_ diffuses through the membrane pores to the gas–liquid interface, it undergoes a chemical reaction with the absorbent flowing in the tube side. As shown in Figure 6, the concentration of the absorbent at the gas–liquid interface decreases significantly due to the substantial consumption by the chemical absorption reaction with CO_2_. In contrast, the absorbent farther from the gas–liquid interface retains a relatively high concentration, as it cannot react with CO_2_ immediately due to the slower diffusion rate of CO_2_. Additionally, under the same operating conditions, the concentration of MEA, PG, and [EMIM][Ac] decreases from 1600 mol/m^3^ to 755 mol/m^3^, 766 mol/m^3^, and 921 mol/m^3^, respectively. This phenomenon reflects the relative CO_2_ removal performance of the absorbents, with MEA exhibiting the best performance, followed by PG, and [EMIM][Ac] showing the lowest performance among the absorbents studied. Furthermore, along the axial direction of the membrane module, the concentration of the absorbent gradually decreases as it moves through the tube side. That is because the gaseous CO_2_ continuously diffuses into the liquid phase and reacts chemically with the absorbent as it progresses along the tube.

### 3.3. Influence of Reaction Heat on CO_2_ Removal Efficiency

**Figure 7:**
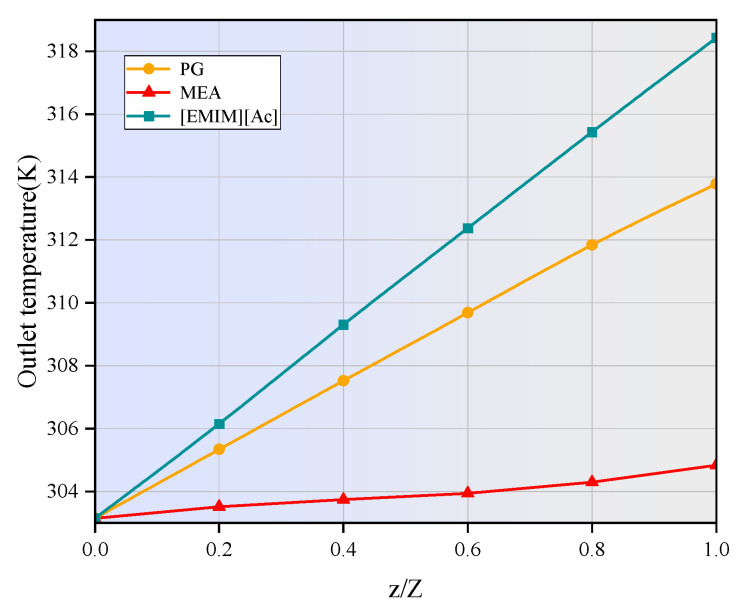
Axial temperature distribution in the tube side. *C_CO2_* = 1 mol/m^3^, *C_L_* = 1600 mol/m^3^, *V_g_* = 0.4 m/s, *V_L_* = 1 m/s, *T* = 303.15 K, *p* = 0.1 MPa.

The reaction between CO_2_ and the studied absorbent is exothermic, leading to an increase in the liquid phase temperature. Figure 7 shows the axial temperature distributions of the three absorbents at an inlet temperature of 303.15 K, with temperature increases ranging from 2 to 15 K at the outlet. The maximum temperatures at the outlet for MEA, PG, and [EMIM][Ac] are 304.8 K, 313 K, and 318 K, respectively. The variation in temperature increase among the different absorbents is influenced by their specific heat capacities and the energy released during the absorption reaction, with reaction enthalpy being the primary contributor of the temperature rise. As the reaction occurs at the gas–liquid interface and the reactants move axially along with the absorbent, the thermal energy released by the chemical reaction gradually accumulates along the length of the membrane contactor, resulting in an overall increase in temperature. The radial temperature gradient in the three absorbents is negligible due to efficient heat dissipation facilitated by the liquid flow.

**Figure 8:**
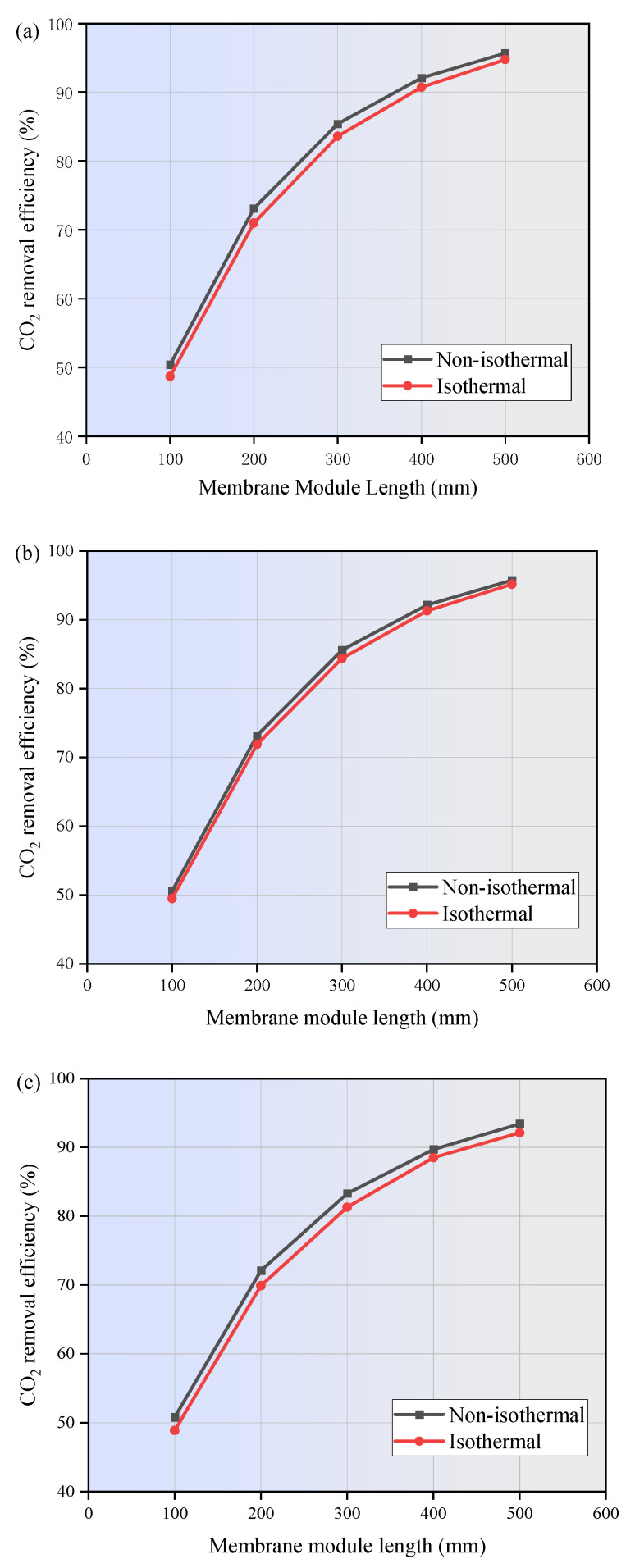
Effect of reaction heat on the absorption properties of (**a**) MEA; (**b**) PG; and (**c**) [EMIM][Ac]. *C_CO2_* = 1 mol/m^3^, *C_L_* = 1600 mol/m^3^, *V_g_* = 0.4 m/s, *V_L_* = 1 m/s, *T* = 303.15 K, *p* = 0.1 MPa.

The effect of reaction heat on the absorption performance of MEA, PG, and [EMIM][Ac] is illustrated in Figure 8. The results indicate that the CO_2_ removal efficiency predicted by the non-isothermal model is superior to that of the isothermal model. The reaction between CO_2_ and the three absorbents is reversible, which releases heat during absorption and absorbs heat during desorption. The increase in temperature along the membrane length intensifies molecular motion in the absorption liquid, leading to a higher frequency of molecular collisions. This enhances the mass transfer rate of CO_2_, allowing it to dissolve more rapidly into the absorption liquid and thereby increase the CO_2_ absorption rate. Additionally, the reaction rate constant increases with temperature, as described by the Arrhenius Equation, further promoting the CO_2_ reaction rate. Figure 8 also demonstrates that the CO_2_ removal efficiency is highly dependent on the membrane module length. As the membrane module length increases from 100 mm to 500 mm, the CO_2_ removal efficiency improves from 50.4% to 95.6% for MEA, from 50.6% to 95.7% for PG, and from 50.8% to 93.2% for [EMIM][Ac], respectively. Longer membrane modules provide extended contact time and a larger surface area, which contribute to a higher CO_2_ removal efficiency.

### 3.4. Effect of Gas Flow Rate and CO_2_ Inlet Concentration on CO_2_ Removal

**Figure 9:**
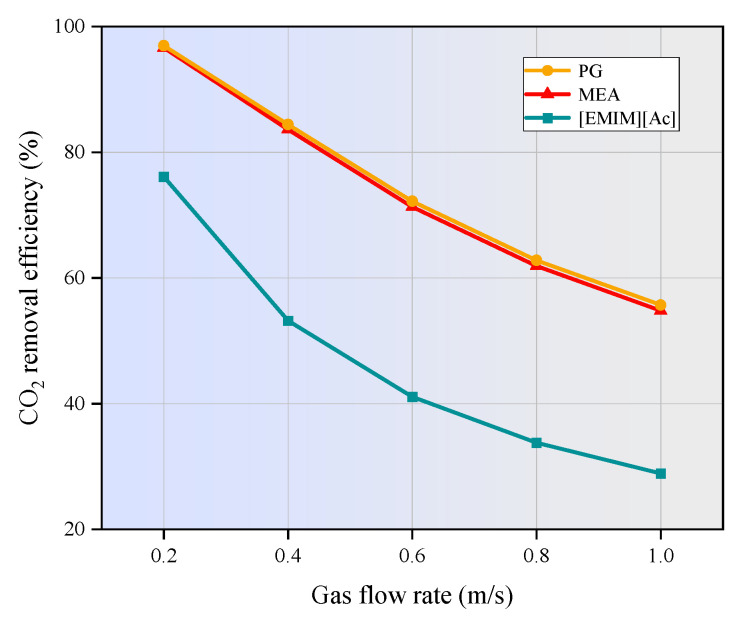
Effect of inlet gas flow rate on CO_2_ removal efficiency. *C_CO2_* = 1 mol/m^3^, *C_L_* = 1600 mol/m^3^, *V_L_* = 1 m/s, *T* = 303.15 K, *p* = 0.1 MPa.

The effect of gas velocity on CO_2_ removal efficiency in the hollow fiber membrane contactor was investigated across a gas velocity range of 0.2 m/s to 1 m/s, with the results shown in Figure 9. As shown, the CO_2_ removal efficiency for all three absorbents decreases as the gas flow rate increases. Specifically, the CO_2_ removal efficiency decreases from 96.6% to 54.8% for MEA, from 96.9% to 55.7% for PG, and from 76.1% to 28.9% for [EMIM][Ac]. At a given liquid phase flow rate, increasing the gas phase flow rate results in a gradual decline in CO_2_ removal efficiency. This is because a higher gas phase flow rate significantly reduces the residence time of the gas within the membrane contactor. As a result, CO_2_ in the flue gas has insufficient time to diffuse into the membrane tube side to react with the absorption liquid. This results in a higher CO_2_ concentration at the outlet of the membrane contactor, ultimately reducing the overall removal efficiency.

**Figure 10:**
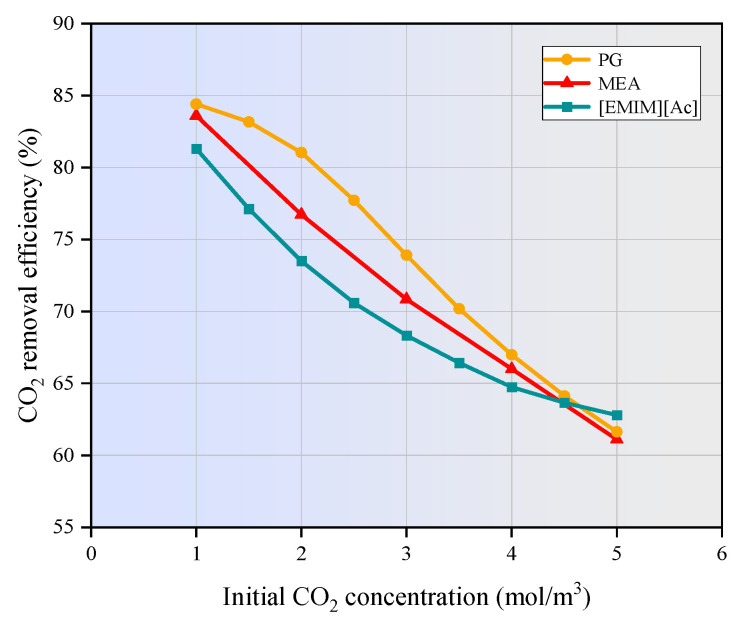
Effect of inlet CO_2_ concentration on absorption properties of the liquid phase. *C_L_* = 1600 mol/m^3^, *V_g_* = 0.4 m/s, *V_L_* = 1 m/s, *T* = 303.15 K, *p* = 0.1 MPa.

Figure 10 illustrates the variation in CO_2_ removal efficiency with changing CO_2_ inlet concentrations.

The liquid phase flow rate was maintained at 1 m/s, the gas phase flow rate at 0.4 m/s, and the CO_2_ concentration in the inlet gas ranged from 1 mol/m^3^ to 5 mol/m^3^. As depicted in Figure 10, the CO_2_ removal efficiency gradually decreases as the CO_2_ inlet concentration increases. Higher CO_2_ concentrations intensify the concentration gradient between the shell and tube sides of the membrane contactor, causing more CO_2_ to diffuse into the liquid phase and react with the absorbent. When the flow rate of the absorption liquid is constant, the absorbent becomes insufficient to fully capture the increased CO_2_ load, resulting in a decrease in CO_2_ removal efficiency.

### 3.5. Effect of Liquid Flow Rate on CO_2_ Removal

**Figure 11:**
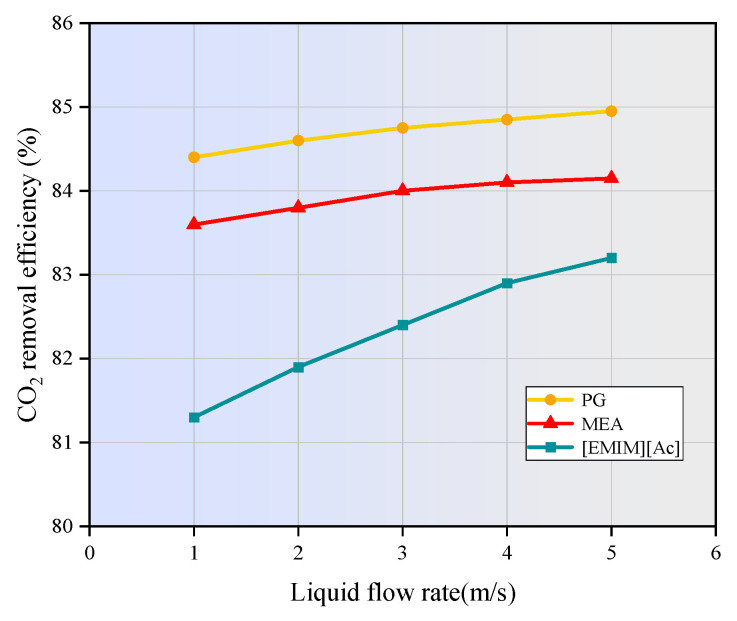
Effect of absorbent flow rate on absorption properties of the liquid phase. *C_CO2_* = 1 mol/m^3^, *C_L_* = 1600 mol/m^3^, *V_g_* = 0.4 m/s, *T* = 303.15 K, *p* = 0.1 MPa.

Figure 11 illustrates the relationship between the CO_2_ removal efficiency and liquid flow rate. As the liquid flow rate increases from 1 m/s to 5 m/s, the CO_2_ removal efficiency gradually increases from 83.6 to 84.1% for MEA, from 84.4% to 84.9% for PG, and from 81.3% to 83.2% for [EMIM][Ac], respectively. A higher liquid flow rate enhances the turbulent disturbance of the absorbent, which allows the reaction products of CO_2_ and the absorbent to diffuse quickly into the bulk liquid and be removed. Additionally, it replenishes the consumed absorbent at the gas–liquid interface more promptly, increasing the CO_2_ concentration gradient between the gas and liquid phases, thereby facilitating the diffusion of CO_2_ into the liquid phase. These factors comprehensively lead to an improvement in CO_2_ removal efficiency.

It can also be observed form Figure 11 that, at a given liquid flow rate, the absorption efficiency of the chemical absorbents PG and MEA is significantly higher than that of [EMIM][Ac]. PG exhibits the highest absorption efficiency, which, according to Yan et al. [41], is due to its strong affinity for CO_2_ and efficient mass transfer properties. MEA is slightly less efficient than PG due to differences in reaction dynamics. In contrast, [EMIM][Ac] demonstrates the lowest absorption efficiency. This is likely because its combination of physical and chemical absorption mechanisms is less effective, and its higher viscosity increases mass transfer resistance.

### 3.6. Effect of Absorbent Concentration on CO_2_ Removal

**Figure 12:**
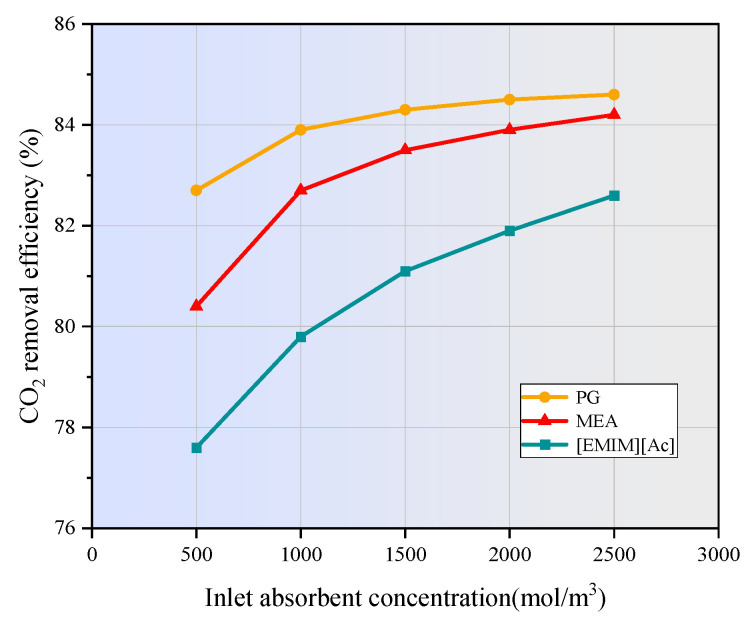
Effect of absorbent concentration on CO_2_ removal efficiency. *C_CO2_* = 1 mol/m^3^, *V_g_* = 0.3 m/s, *V_L_* = 0.1 m/s, *T* = 303.15 K, *p* = 0.1 MPa.

Figure 12 shows the effect of absorbent concentration on CO_2_ removal efficiency. Obviously, increasing the concentration of the chemical absorbent in a hollow fiber membrane contactor significantly enhances CO_2_ removal from the flue gas. Higher absorbent concentrations provide more reactive components, enabling faster and more efficient reactions with CO_2_ as it diffuses through the microporous membrane into the liquid phase. This rapid reaction reduces the CO_2_ concentration on the liquid side, thereby creating a greater concentration gradient that drives additional CO_2_ from the gas phase into the liquid. Consequently, the dissolution rate of CO_2_ in the absorbent increases, leading to an improvement in overall CO_2_ removal efficiency. However, it is important to balance the absorbent concentration with operational factors such as viscosity and potential pressure drop to ensure the optimal performance of the membrane contactor [42]. In contrast to Figure 8, the CO_2_ removal efficiency of [emim][Ac] is significantly lower than that of MEA and PG. This can be attributed to the slower reaction rate of [emim][Ac], and the fact that more stay time is needed to achieve the same efficiency as MEA and PG [43].

### 3.7. Effect of Membrane Porosity on CO_2_ Removal

**Figure 13:**
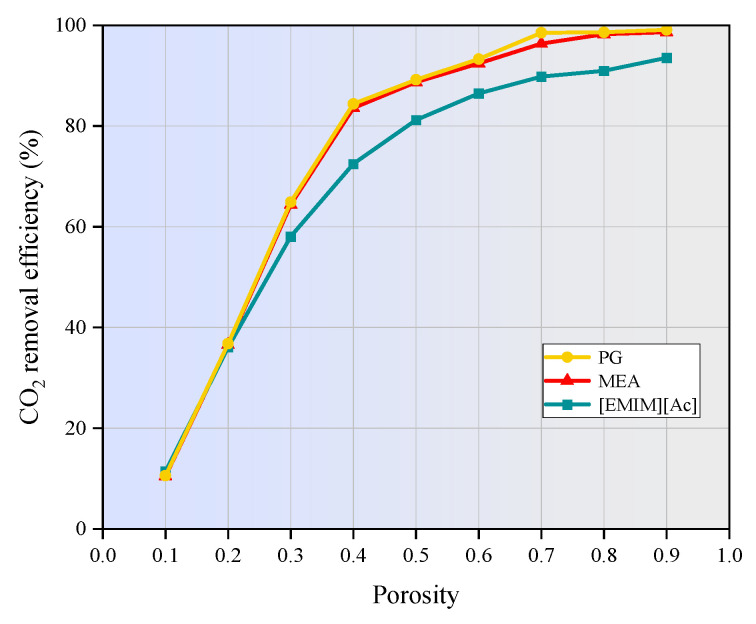
Effect of membrane porosity on CO_2_ removal efficiency. *C_CO2_* = 1 mol/m^3^, *C_L_* = 1600 mol/m^3^, *V_g_* = 1 m/s, *V_L_* = 0.1 m/s, *T* = 303.15 K, *p* = 0.1 MPa.

Figure 13 shows the effect of hollow fiber membrane porosity on the CO_2_ absorption performance of MEA, PG, and [EMIM][Ac] under the non-wetting operation mode. The CO_2_ removal efficiency is enhanced with increasing membrane porosity. Specifically, when the membrane porosity increases from 0.1 to 0.9, the removal efficiency for MEA improves from 10.5% to approximately 95.6%, from 10.6% to 99.1% for PG, and from 11.5% to around 93.5% for [EMIM][Ac], respectively. This enhancement is attributed to the fact that higher porosity results in a greater effective diffusion coefficient of CO_2_ within the membrane pores. Consequently, the mass-transfer resistance is reduced, and CO_2_ diffuses more rapidly and efficiently through the membrane, thereby improving the overall separation efficiency. However, excessively high porosity may decrease the membrane wettability, making it easier for solvents to penetrate the membrane pores, which can increase mass transfer resistance and potentially counteract the benefits of higher porosity. Furthermore, increasing porosity can also compromise the structural integrity of the membrane, reducing its self-supporting ability. This can make membrane fabrication more challenging and affect the long-term stability and durability of the membrane contactor [44]. Therefore, the porosity should be optimized to balance the improvement in CO_2_ removal efficiency with potential challenges related to membrane wettability and structural integrity.

## 4. Conclusions

In this study, the finite element method was applied to numerically investigate the CO_2_ separation by three typical absorbents in a PVDF hollow fiber membrane contactor. The performance of the non-isothermal model was compared with that of the isothermal model. The effects of operating parameters on CO_2_ removal efficiency were studied. The main conclusion can be drawn as follows:

(1) The temperatures along the membrane contactor length for three studied absorbents all show an upward trend, with an increase of 2 to 15 K. This temperature increase intensifies the molecular motion in the absorbent, leading to a higher frequency of molecular collisions. Consequently, the mass transfer of CO_2_ is enhanced, allowing it to dissolve more rapidly into the absorption liquid and thereby increasing the CO_2_ absorption rate.
(2) As the gas flow rate and CO_2_ inlet concentration increase, the CO_2_ removal efficiency decreases significantly. When the gas velocity increases from 1m/s to 5m/s, the CO_2_ removal efficiency of MEA and PG is decreased by 41.8% and 41.2%, respectively. [EMIM][Ac] is more susceptible to the influence of gas velocity, and the corresponding CO_2_ removal efficiency is decreased by nearly 47%. When the CO_2_ inlet concentration increases from 1 mol/m^3^ to 5 mol/m^3^, the CO_2_ removal efficiency of three absorption systems are decreased by around 20%.
(3) The increase in liquid velocity and absorbent concentration has a limited positive effect on CO_2_ removal. When the liquid velocity increases from 1 m/s to 5 m/s, the CO_2_ removal efficiency of MEA, PG, and [EMIM][Ac] is only increased by 0.5%, 0.5%, and 1.9%. While the absorbent concentration increased from 500 mol/m^3^ to 2500 mol/m^3^, the CO_2_ removal rates of MEA, PG, and [EMIM][Ac] is increased by 3.8%, 1.9%, and 5%.
(4) When the membrane length increases from 100 mm to 500 mm, the CO_2_ removal efficiency of MEA, PG, and [EMIM][Ac] is increased from 50% to 95.7%, 95.7%, and 93.2%. When the porosity increases from 0.1 to 0.9, the CO_2_ removal efficiency of MEA, PG, and [EMIM][Ac] is increased by 85.1%, 88%, and 82% respectively.
(5) In this study, PG exhibits the highest absorption capacity, followed by MEA and [EMIM][Ac], and [EMIM][Ac] is more sensitive to changes in various parameters compared to the other two absorbents.

## Figures and Tables

**Table 1:** Specification of the hollow fiber membrane contactor.

Specifications	Value	Unit
Module length (*L*)	100	mm
Module inner radius (*R*)	10	mm
Fiber inner radius (*r*_1_) Fiber outer radius (*r*_2_)	0.16 0.21	mm mm
Porosity (*ε*)	40	%
Tortuosity (*τ*)	4	
Number of fibers (*n*)	600	

**Table 2:** Absorbent properties.

Parameter	Value	Unit	References
*D_CO2-shell_*	1.8 × 10^−5^	m^2^/s	[35]
*D_CO2-MEA_*	1.51 × 10^−9^	m^2^/s	[36]
*D_CO2-PG_*	1.8 × 10^−9^	m^2^/s	[37]
*D_CO2-_*_[EMIM][Ac]_	5.58 × 10^−10^	m^2^/s	[38]
*D_MEA-tube_*	9.32 × 10^−10^	m^2^/s	[36]
*D_PG-tube_*	1.05 × 10^−9^	m^2^/s	[37]
*D*_[EMIM][Ac]-*tube*_	8.36 × 10^−11^	m^2^/s	[38]
*k_MEA_*	$\frac{10^{\left(10.99-2152/T\right)}}{1000}$	mol/m^3^·s	[36]
*k_PG_*	10^16^ exp(−8544/T)	mol/m^3^·s	[37]
*k*_[EMIM][Ac]_	1545 exp(−1240.9/T)	mol/m^3^·s	[38]
*m_CO2-MEA_*	0.86		[39]
*m_CO2-PG_*	0.625		[13]
*m_CO2-_*_[EMIM][Ac]_	0.529		[38]

**Table 3:** Boundary conditions of mass transfer equation.

Boundary	Tube	Membrane	Shell
*z* = 0	$C_{{CO}_{2},tube}=0$ $C_{absorbent,tube}$ *=* $C_{initial}$	Insulated	$\frac{\partial C_{{CO}_{2},shell}}{\partial r}=0$
*z* = *L*		Insulated	$C_{{CO}_{2},,shell}$ *=* $C_{initial}$
*r* = 0	$\frac{\partial C_{{CO}_{2},tube}}{\partial r}=0$		
*r* = *r*_1_	$C_{{CO}_{2},tube}=m_{{CO}_{2}}C_{{CO}_{2},mem}$	$C_{{CO}_{2},mem}=\frac{C_{{CO}_{2},tube}}{m_{{CO}_{2}}}$	
*r* = *r*_2_		$C_{{CO}_{2},mem}=C_{{CO}_{2},tube}$	$C_{{CO}_{2},,shell}=C_{{CO}_{2},mem}$
*r* = *r*_3_			$\frac{\partial C_{{CO}_{2},shell}}{\partial r}=0$

**Table 4:** Boundary condition of energy equations.

Boundary	Tube	Membrane	Shell
*z* = 0	$T_{L,tube}=T_{L,tube-in}$	$\frac{\partial T_{{CO}_{2},mem}}{\partial Z}=0$	$-\lambda_{g,shell}\frac{\partial T_{g,shell}}{\partial Z}=0$
*z* = *L*	$-\lambda_{L,tube}\frac{\partial T_{L,tube}}{\partial Z}=0$	$\frac{\partial T_{{CO}_{2},mem}}{\partial Z}=0$	$T_{g,shell}=T_{g,shell-in}$
*r* = 0	$\frac{\partial T_{L,tube}}{\partial r}=0$		
*r* = *r*_1_	$T_{L,tube}=T_{{CO}_{2},mem}$	$T_{{CO}_{2},mem}=T_{L,tube}$	
*r* = *r*_2_		$T_{{CO}_{2},mem}=T_{g,shell}$	$T_{g,shell}=T_{{CO}_{2},mem}$
*r* = *r*_3_			$\frac{\partial T_{g,shell}}{\partial r}=0$

## Data Availability

The original contributions presented in this study are included in the article. Further inquiries can be directed to the corresponding author.
